# Seismic multi-hazard and impact estimation via causal inference from satellite imagery

**DOI:** 10.1038/s41467-022-35418-8

**Published:** 2022-12-17

**Authors:** Susu Xu, Joshua Dimasaka, David J. Wald, Hae Young Noh

**Affiliations:** 1 Department of Civil Engineering, Stony Brook University, Stony Brook, NY 11790 USA; 2 Department of Computer Science, Stony Brook University, Stony Brook, NY 11790 USA; 3 Department of Civil and Environmental Engineering, Stanford University, Stanford, CA 94305 USA; 4 U.S. Geological Survey, Golden, CO 80401 USA

**Keywords:** Civil engineering, Natural hazards, Computational science, Seismology

## Abstract

Rapid post-earthquake reconnaissance is important for emergency responses and rehabilitation by providing accurate and timely information about secondary hazards and impacts, including landslide, liquefaction, and building damage. Despite the extensive collection of geospatial data and satellite images, existing physics-based and data-driven methods suffer from low estimation performance due to the complex and event-specific causal dependencies underlying the cascading processes of earthquake-triggered hazards and impacts. Herein, we present a rapid seismic multi-hazard and impact estimation system that leverages advanced statistical causal inference and remote sensing techniques. The unique feature of this system is that it provides accurate and high-resolution estimations on a regional scale by jointly inferring multiple hazards and building damage from satellite images through modeling their causal dependencies. We evaluate our system on multiple seismic events from diverse countries around the globe. Our results corroborate that incorporating causal dependencies significantly improves large-scale estimation accuracy for multiple hazards and impacts compared to existing systems. The results also reveal quantitative causal mechanisms among earthquake-triggered multi-hazard and impact for multiple seismic events. Our system establishes a new way to extract and utilize the complex interactions of multiple hazards and impacts for effective disaster responses and advancing understanding of seismic geological processes.

## Abstract (web summary)

This study presents the first rapid seismic multi-hazard and impact estimation system integrating advanced causal inference and remote sensing techniques, which jointly estimates regional-scale and high-resolution maps of seismic multi-hazards and building damage from InSAR imageries.

## Introduction

Moderate-to-large earthquakes are often followed by a series of ground failures and subsequent impacts, such as landslides, liquefaction, and building damage. These cascading hazards and impacts exacerbate seismic losses, including fatalities and disruption to lifelines^1^. For example, the 2018 Hokkaido earthquake in Japan triggered more than 6000 landslides and 1000 associated debris deposits, resulting in more than 80% of the casualties^2^. The series of earthquakes in Ridgecrest, California, in 2019, induced liquefaction in the town of Trona, where one large lateral spread caused severe damage to around 30 square blocks^3^.

Earthquake rapid response systems play an important role immediately after earthquakes in rapidly and accurately locating and estimating the spatial occurrences of seismic hazards (e.g., landslides, liquefaction) and seismic impacts (e.g., building damage). This information serves to direct response and recovery to those areas most in need of critical assistance. Some existing global or regional rapid seismic hazard and impact estimation efforts include GDACS—Global Disaster Alert and Coordination System^4^, PAGER—Prompt Assessment of Global Earthquakes for Response^5^ from the U.S. Geological Survey (USGS), and NERIES-ELER—Network of Research Infrastructures for European Seismology^6^.

Despite the deployment of sophisticated earthquake rapid response systems, the task of accurate and timely localization and estimation of earthquake impacts remains challenging due to uncertainties in data and models, event-to-event variability, and co-occurrence of secondary hazards and impacts. One main difficulty lies in complex causal dependencies underlying the process of earthquake-triggered multi-hazard and impacts. To provide regional earthquake-induced ground failure estimation, previous data-driven approaches^7,8^ utilize statistical or black-box machine learning models to directly fit the complex mapping from designated features to a single hazard to historical inventories. Many of these methods are constrained by the limited availability of ground truth data as well as outdated and coarse-grained geospatial proxies (around 230 m) that entail large uncertainties and low spatial resolutions. Regarding rapid estimations of seismic impacts (e.g., building damage) in a large scale, existing systems often apply fragility functions from a pre-built database (e.g., PAGER^9,10^ and HAZUS^11^). However, it is difficult to gain accurate predictions by applying fragility functions to given ground motion maps. This is mainly because there is no accurate local field information available, such as building type, for most seismic zones and many developing countries that do not have a complete fragility function database. Moreover, these methods mainly focus on modeling a single type of hazard without considering the causal interdependencies among multiple hazards and consequent impacts such as building damage, which led to limited performance and difficulty in physical interpretations.

In recent years, remote sensing observations, such as synthetic aperture radar (SAR) images, have become available within hours to days of an event. SAR can penetrate cloud cover compared to optical satellite images, and provide more complete spatial coverage and higher resolution information about seismic hazards or building damage in various weather conditions^12,13^. Most notably, damage proxy maps (DPMs), developed by researchers of the NASA Advanced Rapid Imaging and Analysis (ARIA) team, extract correlation changes between pre- and post-event images, showing the potential to provide supplementary information for rapid seismic hazard and impact estimation^14^. Recent studies have explored integrating DPMs or other InSAR images with prior geospatial models to estimate or analyze single-type hazard^15–17^ or building damage^18,19^. However, these models (1) all focus on single-type hazards, and (2) they are often trained in a supervised way and need a reasonable amount of ground truth labels for model training—which is not immediately available after earthquakes. One of the key challenges associated with seismic hazard and impact estimation from these remote sensing data is spatially overlapping multiple cascading hazards, building damage, and a large amount of event-specific environmental noise (e.g., changes induced by agricultural activities). This challenge made it difficult for current studies to effectively extract and utilize the information in DPMs for a fast and accurate earthquake-triggered landslide or building damage estimation, let alone predicting multiple hazards and impact simultaneously^18^. For example, direct correlation analysis shows a weak correlation between DPMs and building damage in the 2015 Nepal earthquake^18^. Without considering physical causalities in seismic hazard processes, it is difficult to categorize the types of changes observed from these imagery data, such as ground failures, building damage, and noise from vegetation growth and anthropogenic modifications, especially when these changes co-occur.

To this end, we introduce a new rapid seismic multi-hazard estimation system that effectively integrates single-type hazard geospatial models with rich but noisy information from DPMs, through quantitatively modeling causal dependencies underlying the geological processes and ground surface changes in satellite images. This causal inference model enables the system to jointly estimate multiple hazards and impacts, which then improves the overall accuracy and resolution in regional-scale estimation. We encode the causal dependencies among ground shaking, seismic hazards and impact, as well as ground surface changes captured by satellite images in a causal graph. The causal graph connects and fuses the rich information of event-specific surface change patterns in high-resolution DPMs with event-shared physical insights in geospatial models, through explicitly modeling complex and nonlinear relationships among multiple hazards, building damage, environmental factors, and DPMs. The incorporation of causal dependencies enables comprehensive physical reasoning of the image changes in DPMs as the co-occurrence of multiple seismic hazards and building damage.

## Results

### Causal Bayesian network for modeling seismic multi-hazard and impacts

**Fig. 1:**
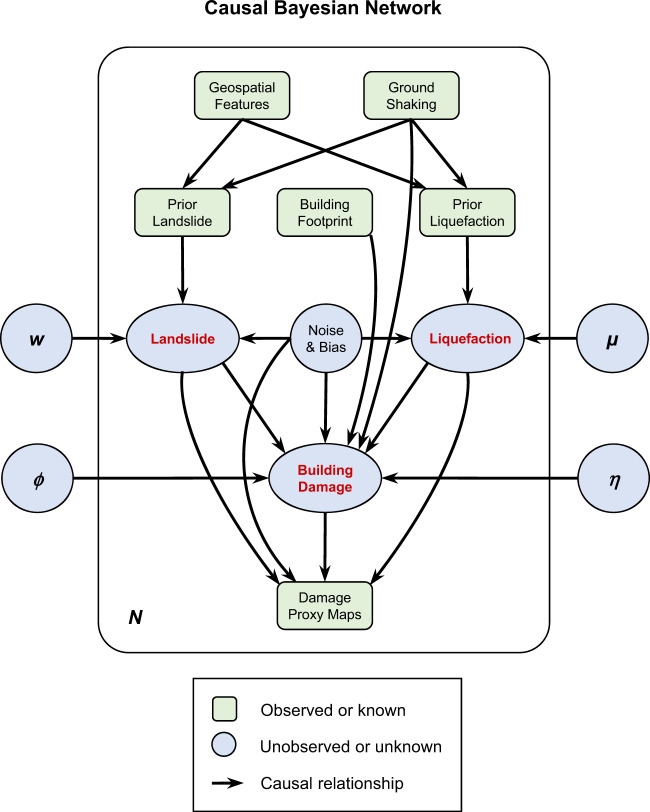
Causal Bayesian network that depicts causal dependencies among different seismic ground failures, building damage, Damage proxy maps (DPMs), and environmental noises. *N* refers to *N* locations/pixels in the target area. The posterior probability of landslide, liquefaction, and building damage at each location are the objectives that our system estimates. Green boxes refer to the variables that have data constraints. Blue circles refer to nodes that are not observed or unknown. *θ*, *ϕ*, *μ*, *η* are the causal coefficients, which are unknown, that quantify the causal effects of parent nodes to landslide, liquefaction, building damage, and DPMs.

The causal graph is formulated as a Bayesian network for encoding a set of conditional dependency relationships, i.e., physical causal relationships, among multiple random variables. Notably, recent advances have shown that causal Bayesian network is a powerful tool for deciphering complex causation among multiple variables from a group of data^20,21^. In our problem, the random variables include unobserved seismic hazards and environmental noise, observed satellite images product (DPMs), each hazard’s geospatial model output, and ground shaking, as well as bias and uncertainties contained in all these observations. The random variables are represented as nodes, while their causal relationships are modeled through edges with directions. An example of our causal graph is shown in Fig. 1. Our model takes prior geospatial models, building footprints, and Damage Proxy Maps for the given earthquake as inputs. The final output of our model includes landslide, liquefaction, and building damage probability maps, as well as causal coefficients between them. The model is further trained in an unsupervised way through Bayesian updating to ensure the model generalization to different countries and regions with a variety of data availability.

The main challenges associated with seismic multi-hazard estimation through the causal Bayesian network are that (1) the ground truth data of multi-hazard and impact are typically not available within weeks to months after an earthquake event; (2) the causal dependencies between multiple hazards, impacts, and remote sensing observations are complex and thus hard to quantify; and (3) the introduced causal Bayesian network has a unique mixture of multiple layers with multiple forms of variables, including binary, log-normal, and logit-normal variables, which makes previous Bayesian inference algorithms no longer applicable. Consequently, it is difficult, if not impossible, to infer the posterior distributions of multiple unobserved variables, especially for events in which multiple seismic hazards and building damage are compounded in a complicated way.

To address these challenges, we derive a novel variational inference algorithm to estimate the posterior distributions of unobserved intermediate seismic hazards, building damage, and their quantitative causal dependencies. Conventional variational inference approximates the posterior distributions of unobserved variables by maximizing the marginal likelihood of observed variables. This has been proven to be a powerful statistical machine learning technique for inferring complex probabilistic models with many unobserved variables^22^. However, existing inference methods assume that intermediate “causal” variables are conditionally independent, which our causal Bayesian network structure violates. For example, both landslide and building damage can cause changes in DPMs, but they are not conditionally independent given DPMs because landslides might be one of the major causes of building damage.

To develop a new variational inference algorithm that works for our problem, we first approximate the likelihood of observed variables by factorizing over the Bayesian network with posteriors of unobserved variables (see Methods). We then derive the lower bound for this approximated likelihood, referred to as variational lower bound, that provides a rigorous theoretical guarantee for the optimality of the joint posterior inference (see Methods). An expectation-maximization algorithm alternatively optimizes the posterior of location-specific multi-hazard and impact together with causal dependencies among them by maximizing the variational bound (see Methods). Furthermore, the stochastic gradient descent and local pruning strategy ensure the scalability and computational efficiency for high-resolution regional multi-hazard and impact estimation. Our model is flexible to incorporate available building footprints, such as those developed by OpenStreetMap^23^ or Microsoft^24^, to further enhance the inference performance over the entire graph by setting the posterior of building damage in those areas without any building footprints to zero. The variational lower bound is generalizable to diverse applications involving discovering causalities among compound hazards.

More importantly, a wealth of experience has been accumulated in recent years in understanding the cascading hazards induced by earthquakes, but our knowledge is still fragmentary. Previously, the causal coefficients and posterior probability could not be learned/optimized simultaneously, owing to the complex causal dependencies. Therefore, most causal coefficients are recovered from correlation analysis using ground truth data of two related variables (e.g., landslide and building damage severity) with the underlying causal direction assumptions^25–27^ (e.g., from landslide to building damage). However, these correlation analyses are often inaccurate because they do not decouple the direct impacts and indirect impacts from shared parents and noise. By jointly optimizing the quantitative causal dependencies over the entire causal Bayesian network, our system provides a systematic way to reveal insights into the event-specific causal mechanisms of compound seismic hazards.

### Evaluation of seismic multi-hazard estimation over multiple seismic events

We evaluate the performance of our rapid multi-hazard and impact estimation strategy using four earthquakes in Puerto Rico (2020)^28^, Hokkaido, Japan (2018)^29^, Ridgecrest, California (2019)^30^, and Central Italy (2016)^31^. These earthquakes occurred in regions with a variety of building structures and seismic, geological, and environmental characteristics. NASA ARIA team generated DPMs using SAR images captured by Japan Aerospace Exploration Agency’s ALOS-2 satellites and European Space Agency’s Sentinel-1 satellites. These DPMs are available within 6–14 days after the earthquakes. We evaluate the predictions of our system using the ground truth data collected by post-event reconnaissance groups, and compare the prediction performance with existing USGS earthquake ground failure products^7,32^ (prior models, see Methods), a regression model that only uses DPMs for ground failure/damage estimation in four different earthquakes, and our model without using building footprint. The ground truth availability is presented in Supplementary Table 3. Furthermore, we show that the introduced system reveals global and regional causality patterns among compound hazards and building damage in post-earthquake settings.

**Fig. 2:**
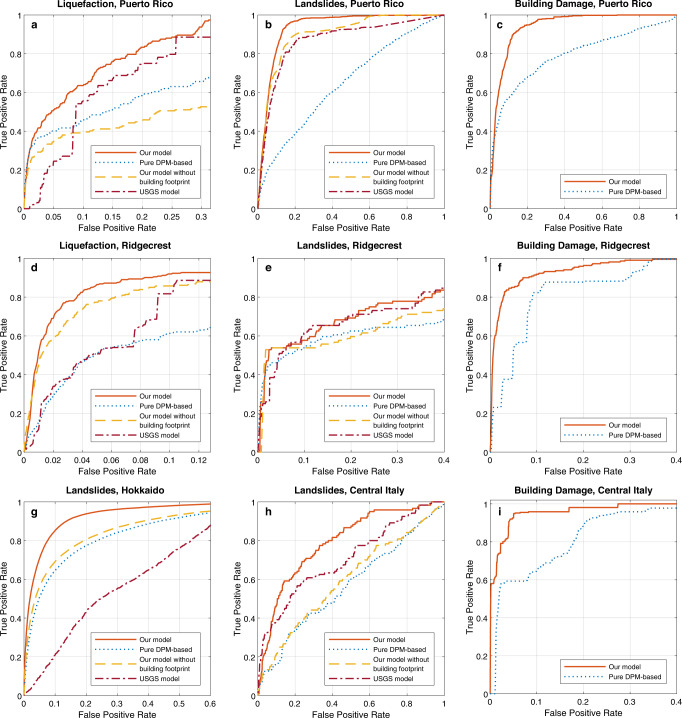
Comparisons of receiver operating characteristic (ROC) curves of four different earthquake events. **a**–**c** Liquefaction, landslides, and building damage ROC curves of the 2020 Puerto Rico earthquake. **d**–**f** Liquefaction, landslides, and building damage ROC curves of the 2019 Ridgecrest earthquake. **g** Landslides ROC curve of the 2018 Hokkaido, Japan earthquake. **h**, **i** Landslides and building damage ROC curve of the 2016 Central Italy earthquake.

We summarize the performance of our system and baseline methods using the receiver operating characteristic (ROC) curve, which visualizes how the true positive rate varies with false positive rate (see Methods). We also calculate the area under the ROC curve (AUC)^33^, representing the ability of a system to distinguish the positive and negative cases, and cross-entropy loss^34^, measuring the distributional dissimilarity between predictions and ground truth data (see Methods). Both evaluation metrics are used to evaluate the disaster impact estimation system’s ability to identify ground failures and to accurately estimate their probabilities compared to the binary ground truth. Figure 2 presents the performance evaluation results, including the ROC curves of our system (orange solid line), existing USGS model (red dotted line), pure DPM-based indicator (blue dotted line), and our model without using building footprint (yellow dotted line). In Fig. 2a, d, we compare the binary liquefaction estimation performance of our system with baseline methods for the Puerto Rico and the Ridgecrest earthquakes, respectively. These two earthquakes induced substantial liquefaction and subsequent building damage in the target estimation areas. Figure 2b, e, g, h compares the landslides occurrence estimation performance of our system with baseline methods for the Puerto Rico earthquake, the Ridgecrest earthquake, the Hokkaido earthquake, and the Central Italy earthquake, respectively. Figure 2c, f, i presents the ROC curves for binary building damage estimates (no/slight damage versus moderate/severe/collapse damage) in the Puerto Rico earthquake, the Ridgecrest earthquake, and the Central Italy earthquake. The Hokkaido earthquake does not have building damage ground truth available.

As Fig. 2 shows, our system significantly improves the landslide and liquefaction prediction ability compared to the USGS models. It can be seen that our model effectively integrates the information from building footprints, prior models, and DPMs, and significantly improves the ground failure estimation performance compared to traditional geospatial models and pure DPMs-based models. One most recent study explored updating the empirical landslide models progressively using landslide indicators derived from InSAR images to this model in the order they were acquired following the earthquake^15^. But when comparing the landslide estimation performance in the 2018 Hokkaido earthquake, our model achieves the AUC value of 0.93, which significantly improves the landslide estimation performance compared to their DPM-based model^15^ which achieves 0.78 AUC. The comparison also shows that the integration of building footprints contributes to the performance improvement of our system significantly, especially for predicting landslides in the 2016 Central Italy earthquake and predicting liquefaction in the 2020 Puerto Rico earthquake. This is mainly because they mainly occurred in the areas with many buildings. The building footprint information directly improves building damage estimation from DPMs, and indirectly improves liquefaction and landslide estimations. Our model significantly improves the predictions compares to USGS models or DPMs alone. In our system, the building footprint provides information about buildings’ existence in different locations, to further reduce the uncertainty in the process of attributing DPM data to landslide/liquefaction/building damage.

We also show that even without the prior fragility function, the model can still provide meaningful predictions about the building damage distribution. We compare the performance for building damage detection (no/slight building damage versus moderate/severe/collapse building damage), between our model and the pure DPM-based method in Fig. 2c, f, i. The results show that our system improves the binary building damage detection accuracy by upto 16.92%. Another recent work G-DIF proposes to train a kriging regressor that combines geospatial models with InSAR images for building damage estimation using inventory data and ground truth, but the supervised method performance is constrained on ground truth data availability^18^. Our results show that by integrating information from building footprints, DPMs, and causal ground failure factors, it is possible to estimate large-scale building damage distributions in an accurate and rapid manner, even without prior fragility function and ground truth data.

More detailed information about the ground truth data availability can be found in Supplementary Table 3. Note that the ultimate goal of performance comparison is to show our model’s capability to improve the estimation performance of multi-hazards and damage by jointly modeling them and integrating geospatial models with DPMs. Therefore, even though there are only one or two types of ground truth observations, the performance improvement can still indicate the capability of our model. In the meantime, in most cases, if the ground truth data of one ground failure is not available, it usually indicates that that ground failure is not very critical so the survey agency deprioritizes the investigation of that ground failure. For example, in the Hokkaido earthquake, although liquefaction ground truth is not available, we still show that by integrating multiple hazards and damage with DPMs, we can significantly improve the performance of landslide prediction—which is the most critical ground failure in the covered area^35^.

In the following sections, we present detailed results in the 2020 Puerto Rico earthquake and the 2018 Hokkaido earthquake, which have different ground failure and damage patterns. The Puerto Rico earthquake-induced landslides and severe liquefaction, resulting in damage to residential and commercial structures and human fatalities, according to the reconnaissance reports from multiple groups^36^. The Hokkaido earthquake mainly triggered numerous shallow landslides, especially in the towns of Atsuma and Abira, and subsequent building damage^37,38^. We present the detailed evaluation results of our system on these two distinct types of earthquake events. More evaluation results about the 2019 Ridgecrest earthquake and the 2016 Central Italy earthquake are presented in the “Methods” section.

#### The 2020 Puerto Rico Earthquake

On January 7, 2020, at 4:24 a.m. (AST), a magnitude 6.4 earthquake shook the southwest part of Puerto Rico^28^. Following the disaster, post-disaster reconnaissance efforts revealed widespread casualities with over 775 affected buildings^39^ and 800 ground failure observations^40,41^. To locate potentially damaged areas, the ARIA team created DPMs using SAR images from the Sentinel-1 satellite^42^. Ground truth observations were later collected in field reconnaissance conducted by researchers from the USGS, the University of Puerto Rico Mayagüez, the GEER team and the Structural Extreme Events Reconnaissance team^36,43,44^.

**Fig. 3:**
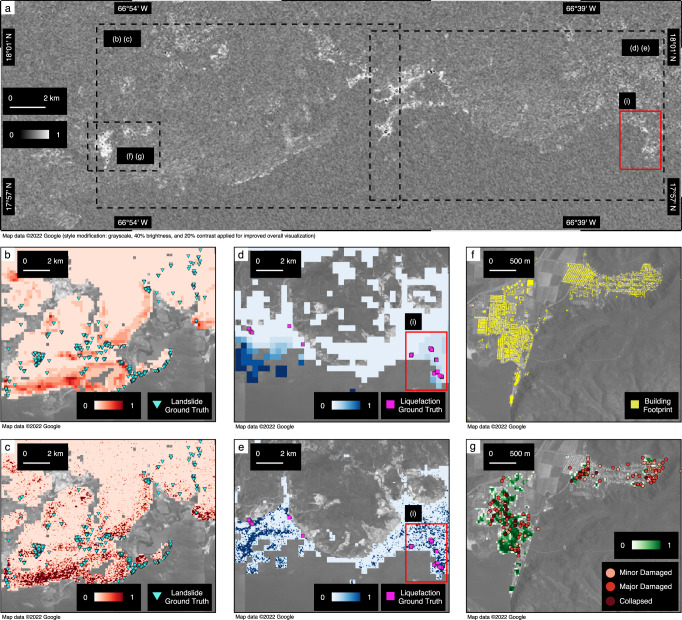
Geospatial prior and posterior estimation models of the 2020 Puerto Rico earthquake. **a** Damage proxy map (30-m resolution) with rectangular extents of each focused area. **b** Prior landslide estimates in red and ground truth (triangles). **c** Posterior landslide estimates in red and ground truth (triangles). **d** Prior liquefaction estimates in blue and ground truth observations (squares). **e** Posterior liquefaction estimates in blue and ground truth (squares). **f** Building footprint map. **g** Posterior building damage estimates in green and ground truth (rounded points). Rectangle **i** highlights the area of western Ponce.

We compared our system performance with benchmark methods for estimating ground failures and building damage occurrence in the Puerto Rico earthquake in Figs. 2a–c and  3. The AUC for our liquefaction ROC curve is improved by 15.64% and cross-entropy loss is reduced by 52.45% compared to the existing model. In landslide estimation, our model improves the AUC by 7.40% and reduces the cross-entropy loss by 51.57%. In building damage estimation, our model improves the AUC by 33.14% and reduces the cross-entropy loss by 44.34%. As an example, when our system performance converges as false positive rate increases to 31.80%, our system achieved a 97.40% true positive rate for liquefaction estimates compared to an 88.54% true positive rate of the USGS liquefaction model, as shown in Fig. 2a. Figure 3 visualizes our model performance and ground truth observations in the shaken region for joint landslide (Fig. 3c), liquefaction (Fig. 3e), and building damage estimation (Fig. 3g), compared with the prior landslide model (Fig. 3b) and prior liquefaction model (Fig. 3d), and building footprint (Fig. 3f). In Fig. 3a, the rectangle (i) covers the western areas of Ponce, which is the second largest city in Puerto Rico and suffered from severe liquefaction and lateral spreading during the earthquake^36^(magenta squares in Fig. 3d, e). Our system predicts a high probability of liquefaction in these areas (Fig. 3e(i)) compared to the existing USGS liquefaction model (Fig. 3d(i)), showing that integrating DPMs through our causal Bayesian network helped improve the accuracy of rapid liquefaction identification.

#### The 2018 Hokkaido, Japan Earthquake

**Fig. 4:**
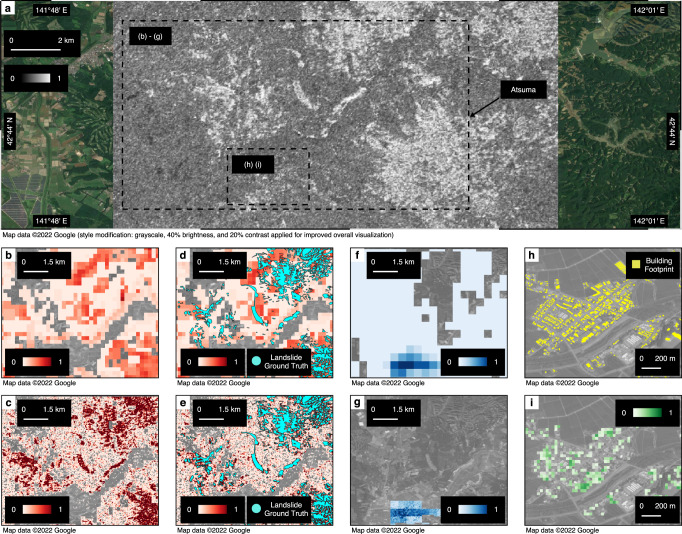
Geospatial prior and posterior estimation models of the 2018 Hokkaido, Japan earthquake. **a** Damage proxy map (30-m resolution) with rectangular extents of each focused area. **b** Prior landslide estimates. **c** Our landslide estimates. **d** Prior landslide estimates (in red) with ground truth observations (in blue polygon). **e** Our landslide estimates (in red) with ground truth observations (in blue polygon). **f** Prior liquefaction estimates. **g** Our liquefaction estimates. **h** Building footprint map. **i** Our building damage estimates. The quantitative performance comparison results could be found in Fig. 2.

On September 6, 2018, at 3:08 a.m. (JST), two days after Typhoon Jebi made landfall with heavy rain, a Mw 6.6 earthquake caused catastrophic landslides in the southern area of Hokkaido, Japan^29,45^. Using the SAR images from the Japan Aerospace Exploration Agency’s ALOS-2 satellites, the ARIA team produced DPMs^46^ that includes the town of Atsuma, which was located near the sites of large-scale landslides^47^. Figure 4a presents the target seismic zone and corresponding DPM. This DPM covered the towns of Atsuma and Abira. Ground truth observations of landslides were later collected by the Geospatial Institute of Japan^47^. The liquefaction investigation and building damage reports do not cover the extent of the DPM. With the current ground failure hazard system of the USGS, the prior estimations of landslide and liquefaction probabilities were generated using the corresponding ShakeMap in Atlas V4^29^.

We present our model performance for joint landslide, liquefaction, and building damage estimation in Fig. 4, compared with the USGS landslide models and USGS liquefaction models, as well as ground truth inventories^7,23,24,32,48^. The results show that our landslide model (Fig. 4c) resembles the spatial distribution of ground truth observations (Fig. 4e) more accurately than the USGS models (Fig. 4a). The posterior model also has a higher resolution (30 m) than the USGS model (230 m)^29^. Our model, by integrating the DPM and prior models (Fig. 4a), identifies more landslide occurrences that are later validated by ground truth observations, compared to existing USGS models. The AUC is improved by 42.02%, achieving 0.9331, and the cross-entropy is reduced by 47.75%. In addition, the liquefaction and landslide estimation map resolutions are improved by 7.7 and 15 times, respectively. The results demonstrate that the joint estimation of landslide and liquefaction using high-resolution satellite images significantly improves the landslide estimates.

### Revealed causality relationships

**Fig. 5:**
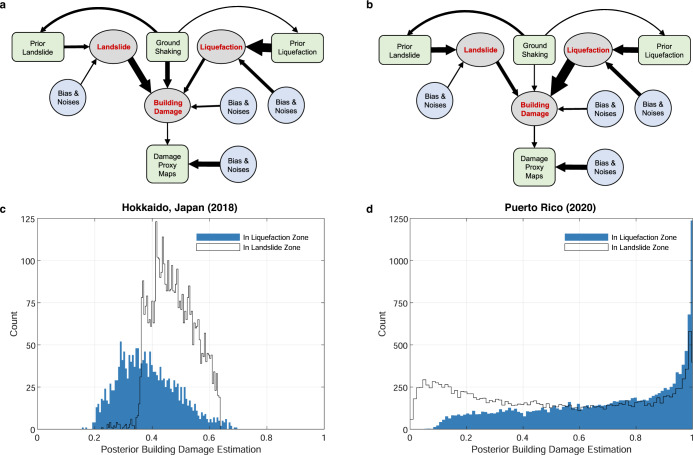
Analysis of causal mechanisms revealed by the graph. **a** Visualization of quantified causal graph for the 2018 Hokkaido, Japan earthquake. The thickness of each edge represents quantified importance of causal effect over the edge. **b** Visualization of quantified causal graph for the 2020 Puerto Rico earthquake. The thickness of each edge represents quantified importance of causal effect over the edge. **c** Estimated building damage probability distribution under the landslide zone (transparent white) and the liquefaction zone (colored blue) for the 2018 Hokkaido, Japan earthquake. **d** Estimated building damage probability distribution under the landslide zone (transparent white) and the liquefaction zone (colored blue) for the 2020 Puerto Rico earthquake.

The inferred causal graphs for each earthquake site revealed the causal dependencies between ground shaking, landslide, liquefaction, building damage, and environmental noise. In Puerto Rico and Ridgecrest, California (see Methods), the building damage is mainly caused by liquefaction rather than landslides. The quantified causal coefficient from liquefaction to building damage for Puerto Rico and Ridgecrest, California are 1.718 (±0.116) and 2.059 (±0.151), respectively, which is significantly higher than the coefficient from landslide to building damage (0.527 ± 0.305 and 1.560 ± 0.374, respectively). In the regions covered by DPMs in the Hokkaido, Japan earthquake and the Central Italy earthquake (see Methods), the building damage is mainly dominated by landslides rather than liquefaction. The quantified causal coefficient from landslides to building damage for the Hokkaido, Japan earthquake and the Central Italy earthquake are 0.848 (±0.128) and 0.966 (±0.575), respectively, which are significantly higher than the coefficient from liquefaction to building damage (0.432 ± 0.161 and 0.345 ± 0.157) in pairwise comparisons. We further enhance the causal graph using the absolute value of coefficients as the linewidth to depict different importance levels in the causal relationships in Fig. 5a, b. This enhanced causal graph visualization reveals that most building damages are directly dominated by the landslide and seismic ground shaking in the 2018 Hokkaido, Japan earthquake, whereas by liquefaction in the 2020 Puerto Rico earthquake. Moreover, Fig. 5 shows that the weight ratio of bias to landslide divided by prior landslide to landslide is higher in the Hokkaido event compared to the Puerto Rico event. This indicates that the prior landslide model in the Puerto Rico event contributes more information to the posterior landslide estimation than the noise/bias term, compared to Hokkaido event, which could be further validated by comparing the AUC of ROC curves of prior landslide models for the Hokkaido event (0.69) and the Puerto Rico event (0.90).

The quantified causal relationships, in conjunction with spatial distribution of buildings, determine the building damage patterns in seismic zones. By investigating the spatial distributions of building footprints, we found that in Ridgecrest, California, more than 80.17% of buildings are located in liquefaction zones, whereas in Puerto Rico, Hokkaido, and Central Italy, 61.87%, 68.39%, and 90.25% of buildings are located in the potential landslide zones, respectively. Interestingly, in the Puerto Rico earthquake, though most buildings are located in the landslide zones, the major building damage are caused by liquefaction. From the distributions in Fig. 5d, we found that building damage distribution in liquefaction zones are skewed towards higher probability than in landslide zones, which indicates more severe building damage in liquefaction zones. Compared to Puerto Rico, as shown by Fig. 5c, in Hokkaido most severe building damage happened in the landslide zones, which matches our final causal graph that landslide is the major cause of building damage in the towns of Atsuma and Abira^37^. Moreover, the revealed causalities in the 2019 Ridgecrest, California earthquake are consistent with the ground truth observations^3^. The significant effects of liquefaction on building damage are also shown in Trona-Argus area of Ridgecrest where heavily damaged buildings were located on or near the alluvial fan and exhibited evidence of lateral spreading in the form of tension cracks. In addition, the building damage observed in Argus had the form of chimney separations from buildings, masonry cracks from extensional movements, toppled walls, and punch-through in retention walls, which are commonly observed types of damage induced by liquefaction^3^.

The causal dependencies revealed in our system contribute to the quantitative explanation of the underlying mechanisms in cascading seismic hazards and impacts, providing a rapid and quantitative idea of potential dominant causations of building damage in different earthquake events from data perspectives. When modeling the causal graph, we incorporated prior understanding of causal dependencies in underlying hazards processes. For example, landslides and liquefaction may induce building damage. The probabilistic inference based on DPMs helps quantify this causal dependencies. This is similar as using causal Bayesian network quantify the causal dependencies between diseases and different signs and symptoms, which is commonly used for human disease modeling and diagnosis^49,50^.

## Discussion

In this study, we show that by jointly estimating multiple seismic hazards and losses considering their causal dependencies, the accuracy and resolution of rapid seismic multi-hazard and impact estimations are significantly improved. In addition, variational inference allows us to guarantee scalability and computational efficiency. This is a holistic work enabling joint estimation of earthquake-induced landslide, liquefaction, and building damage, without the need of any ground truth observations. We demonstrated that DPMs provide effective information related to various types of seismic hazards and impacts, and developed a systematic approach to extract and utilize such information. Meanwhile, causal Bayesian network we developed is a novel multi-layer network that can incorporate a mixture of different data types, including binary log-normal, and logit-normal variables. We derived a tight variational lower bound for this type of Bayesian network, which can be further generalized to different applications involving discovering causalities among other compound hazard and impact distributions.

In addition, our system quantified the causal effects from multiple triggering factors to building damage. We found that the earthquake-induced building damage was mainly caused by landslides in the epicentral areas of 2018 Hokkaido, Japan earthquake and 2016 Central Italy earthquake, while liquefaction were the main contributors in the 2020 Puerto Rico earthquake and 2019 Ridgecrest earthquake. Among four earthquake events, ground shaking is always the most critical causal factor, directly or indirectly, on the occurrences of building damage. Furthermore, we inspected the estimates of building damage distributions in seismic zones with potential landslides and liquefaction separately. The building damage distribution patterns differ significantly, because the underlying earthquake-triggered geophysical impacts vary from event to event. The discovered causal dependencies need to be further combined with the domain knowledge and previous geological survey data to derive rigorous conclusions about local geo-hazards and impacts causal mechanisms. The geologists and engineers could use these quantitative causal measurements as auxiliary information to further guide their field survey and geological analysis.

The solution we developed establishes a new way to understand the complex interaction of multiple hazards from high-resolution satellite signals. The causal Bayesian network has the potential to be applied to other extreme disasters such as hurricanes and flooding through modeling the causalities among their cascading hazards and impacts. One major advantage of our proposed causal Bayesian network is to formulate heterogeneous variables, such as hazards, impacts, and sensing data, into structured Bayesian representations and integrate them into one holistic model informed by physical causality. Therefore, it is flexible to add nodes to or delete nodes from the causal graph according to disaster types. Recently, emerging technology has enabled the use of satellite imagery to identify the conditions of remote areas not easily accessible by disaster responders. The revisit time frequency of satellites has been reduced in recent years from weeks to several days and even to hours^51^. Flexible SAR systems, such as airborne SAR^52^, are being deployed to acquire high-resolution images in a much more timely and controllable way. The developments in the SAR arena make the acquisition of large-scale high-resolution SAR images faster (within hours) and cheaper in the future^53^, offering even greater potential for our computational strategies as these advancements come online. Depending on the timeline of emergency responses and rehabilitation of specific events, the outcome of our system can be combined with the initial loss estimation, loss updating, and/or rehabilitation planning tools to provide situational awareness to broader users in the critical early hours to days, prior to complete observations can be collected. Causality-informed multi-hazard and impact modeling like our variational Bayesian inference system will address the need for rapid and accurate disaster-induced multi-hazard and impact estimation, even without prior models for all subsequent hazards and impact.

## Methods

### Additional data

#### The 2016 Central Italy Earthquake

On August 24, 2016, at 3:36 a.m. (CEST), a Mw 6.2 earthquake resulted in over 298 deaths, 386 injured, 5000 displaced individuals, and many damaged buildings in the Arquata del Tronto-Accumoli-Amatrice area^31,54^. To map the damage over the large affected area, the ARIA team produced DPMs^55^ using the SAR images from the Italian Space Agency’s COSMO-SkyMed satellites and Japan Aerospace Exploration Agency’s ALOS-2 satellites within two days. The landslide inventories indicated a limited area exposed to landslides and no significant liquefaction^41,48^.

#### The 2019 Ridgecrest Earthquake

On July 5, 2019, at 8:19 p.m. (PST), the largest event in the sequence, with a magnitude of 7.1, caused significant ground surface displacements^56^ in the Indian Wells and Searles Valleys. In the towns of Ridgecrest and Trona^30^ extensive field mapping revealed many damaged buildings, landslides, and liquefaction^17,57–59^. After seven days, the ARIA team generated DPMs^60^ using the Sentinel-1 SAR images to identify affected areas.

### The joint natural hazards estimation workflow

The variational Bayesian causal inference system we introduced integrates geospatial models, multiple unobserved ground failures, unobserved building damage, Damage Proxy Maps (DPMs), and their causal dependencies for the joint estimation of multiple unobserved seismic hazards and impacts. Our system first formulates a causal Bayesian network based on the understanding of potential causal relationships underlying the geological processes, as shown in Fig. 1. In Fig. 1, all green rectangle nodes, including geospatial models, building footprints, and DPMs are already observed (known). Our objective is to use this information and this causal dependency relationships represented by the causal network to infer the unobserved variables—mainly landslide, liquefaction, and building damage maps, as well as the causal coefficients between them. Note that each edge in the graphical model indicates a causal relationship but not the input-output data flow. A variational bound is further derived to approximate the intractable (i.e., cannot be directly modeled) posteriors of unobserved ground failure and building damage using the likelihood of observed DPMs. Finally, a stochastic coordinate descent method is employed to find the optimal coefficients representing the causal dependencies among different predictors, ground failure, building damage, environmental and anthropogenic noise, and DPMs. The resulting combinations of causal coefficients and posteriors of unobserved variables are near-optimal to maximize the marginal likelihood of observed DPMs given prior geospatial models. Our system is built upon this causal graphical model to output the probability of intermediate hazards and impacts at each high-resolution grid cell using the geospatial models and DPMs.

#### Causal graph-based Bayesian network

We model a causal graph-based Bayesian network to represent the causal dependencies among landslide (LS), liquefaction (LF), building damage (BD), and DPMs, as shown in Supplementary Table 1. We denote each location in the shaken area as *l*. *y*^*l*^ refers to the DPM pixel captured at *l*. Given a node *i*, we use ${{{{{{{\mathcal{P}}}}}}}}(i)$ to define the parents of *i*. For notational simplicity, we define unobserved seismic ground failure and impact nodes as *x*_*i*_, where *i* ∈ {1, 2, 3}. *x*_1_ refers to landslide (LS), *x*_2_ refers to liquefaction (LF), and *x*_3_ refers to building damage (BD). These unobserved nodes have binary variables *x*_*i*_ ∈ {0, 1}. We define a bias node *x*_0_, which is always active (*x*_0_ = 1), which allows its child nodes to be active even when other parent nodes are inactive. For example, even though landslide and liquefaction do not occur, it is still possible to have building damage. All nodes are linked by an arbitrary directed acyclic graph as shown in Supplementary Fig. 1. We further give quantitative definitions of these links, i.e., causal dependencies, among different random variables. To simplify the notation, we define and *w*_*ki*_ (*k* is any parent node of *i*) to quantify the causal effects from the noise term to a node *i* as well as from a parent node *k* to a child node *i*. When *i* refers to LS, LF, BD, DPM, there is *w*_*ki*_ = *θ*_*k*_, *μ*_*k*_, *ϕ*_*k*_, *η*_*k*_, respectively. All above weight nodes *w*_*ki*_ are formulated as deterministic variables. We assume the formulation of underlying causal dependencies from parent nodes to unobserved *x*_*i*_ and DPM (*y*) as follows:

1 $$\log \frac{p({x}_{i}=1|{x}_{{{{{{{{\mathcal{P}}}}}}}}(i)},{\epsilon }_{i})}{1-p({x}_{i}=1|{x}_{{{{{{{{\mathcal{P}}}}}}}}(i)},{\epsilon }_{i})}={w}_{0i}+{w}_{{\epsilon }_{i}}{\epsilon }_{i}+\mathop{\sum}\limits_{k\in {{{{{{{\mathcal{P}}}}}}}}(i)}{w}_{ky}{x}_{k}$$

2 $$\log y={w}_{\epsilon }{\epsilon }_{y}+{w}_{0y}+\mathop{\sum}\limits_{i\in {{{{{{{\mathcal{P}}}}}}}}(y)}{w}_{iy}{x}_{i}.$$

As *ϵ*_*y*_ is a normal distribution, $y|{{{{{{{\mathcal{P}}}}}}}}(y)$ is subject to a log-normal distribution.

The above logit relationship between LS/LF/BD and their parent nodes follows the assumption of a logistic regression model, which is used in the statistical models of LS and LF used by the USGS^7,32^. With the above distribution and conditional distribution assumptions, we construct a Bayesian network based on the causal graph that effectively captures the dependencies between different ground failure types, building damage, and remote sensing observations. This network contains multiple unobserved random variables and unknown causal dependencies between random variables. The complex causal dependencies make the posterior of unobserved random variables intractable. Therefore, we develop a stochastic variational inference method to approximate the intractable posterior of unobserved ground failure and building damage.

#### Variational causal Bayesian inference

With the Bayesian network constructed, we further infer the posterior of ground failure and building damage. However, the problem is that (1) both causal dependencies and distributions of ground failures and building damages are unknown, and (2) the target affected area could be large, which makes it computationally expensive to jointly update the posteriors of ground failure/building damage over the entire map with the size ranging from 12.72 km × 17.34 km (2018 Hokkaido, Japan earthquake) to 78.15 km × 177.06 km (2019 Ridgecrest, CA earthquake). To address this problem, the variational inference is introduced to factorize the Bayesian network first and then approximate the posterior distributions of unobserved variables through maximizing the log-likelihood of observed variables. To ensure the scalability of our method, the variational inference is conducted on a small batch of randomly sampled locations in each iteration. For each location *l*, we define a variational distribution *q*(*X*^*l*^), which further factorizes over unobserved nodes:

3 $$q({X}^{l})=\mathop{\prod}\limits_{i}q({x}_{i}^{l})=\mathop{\prod}\limits_{i}{({q}_{i}^{l})}^{{x}_{i}^{j}}{(1-{q}_{i}^{l})}^{1-{x}_{i}^{l}}.$$

Given a map containing a set of locations *N*, we further derive a tight lower bound for the log-likelihood as follows:

4 $$\log P(Y)=	\,\mathop{\sum}\limits_{l\in N}\log P\left({y}^{l}\right)\\ \ge 	\mathop{\sum}\limits_{l\in N}\left\{-\log {y}^{l}-\log {w}_{{\epsilon }_{y}}-\frac{{(\log {y}^{l})}^{2}+{w}_{0y}^{2}+{\sum }_{k\in {x}_{{{{{{\mathcal{P}}}}}}({y}^{l})}}{w}_{ky}^{2}{q}_{k}^{l}}{2{w}_{{\epsilon }_{y}}^{2}}\right.\\ 	-\frac{{\sum }_{\begin{array}{c}i,j\in {x}_{{{{{{\mathcal{P}}}}}}(y)}\\ i\ne j\end{array}}2{w}_{iy}{w}_{jy}{q}_{i}^{l}{q}_{j}^{l}-2{w}_{0y}\log {y}^{l}-2(\log {y}^{l})({\sum }_{k\in {x}_{{{{{{\mathcal{P}}}}}}(y)}}{w}_{ky}{q}_{k}^{l})}{2{w}_{{\epsilon }_{y}}^{2}} \\ 	-\frac{2{w}_{0y}{\sum }_{k\in {x}_{{{{{{\mathcal{P}}}}}}({y}^{l})}}{w}_{ky}{q}_{k}^{l}}{2{w}_{{\epsilon }_{y}}^{2}}\\ 	-\mathop{\sum}\limits_{\begin{array}{c}i\in \{{{{{{\rm{LS}}}}}},{{{{{\rm{LF}}}}}}\}\\ \nu \in \{0,1\}\end{array}}{\left({q}_{i}^{l}\right)}^{\nu }{\left(1-{q}_{i}^{l}\right)}^{1-\nu }\log \left(1+\exp \left({(-1)}^{\nu }({w}_{0i}+{w}_{{\alpha }_{i}}{\alpha }_{i})+\frac{{w}_{{\epsilon }_{i}}^{2}}{2}\right)\right)\\ 	-\mathop{\sum}\limits_{\begin{array}{c}{\nu }_{i},{\nu }_{j}\in \{0,1\}\\ i\in \{{{{{{\rm{BD}}}}}}\},j\in {{{{{\mathcal{P}}}}}}(i)\end{array}}\log \left\{1+\exp \left[{(-1)}^{{\nu }_{i}}\cdot \left({w}_{0i}+\mathop{\sum}\limits_{j\in {{{{{\mathcal{P}}}}}}(i)}\frac{1-{(-1)}^{{\nu }_{j}}}{2}{w}_{ji}\right)+\left.\frac{{w}_{{\epsilon }_{i}}^{2}}{2}\right)\right]\right\} \\ 	\hskip 15pt \mathop{\prod}\limits_{k\in \{i,j\}}{\left({q}_{k}^{l}\right)}^{{\nu }_{k}}{\left(1-{q}_{k}^{l}\right)}^{1-{\nu }_{k}}\\ 	-\left.\mathop{\sum}\limits_{i\in \{{{{{{\rm{LS,LF,BD}}}}}}\}}\left[{q}_{i}^{l}\log {q}_{i}^{l}+\left(1-{q}_{i}^{l}\right)\log \left(1-{q}_{i}^{l}\right)\right]\right\}.$$

With the tight lower bound of log-likelihood of DPM observations, we can further maximize the lower bound to find optimal posteriors of unobserved variables, i.e., LS, LF, and BD.

Our final objective is to maximize the bound to find optimal combinations of posteriors and causal dependencies estimations. As both posteriors and weights are unknown, we develop an expectation-maximization approach to achieve this. In the step of expectation, we derive closed-form update equations for local posteriors of LS, LF, and BD, i.e., ${q}_{i}^{l},i\in \{1,2,3\},l\in N$, by maximizing the lower bound and setting the gradients of the lower bound as 0, i.e.,$\partial L({q}^{l},w)/\partial {q}_{i}^{l}=0$. This gradient is obtained via the chain rule, and the optimal posterior follows the form below:

5 $${q}_{i}^{l}=\frac{1}{1+\exp \left(-T({q}_{{{{{{{{\mathcal{P}}}}}}}}(i)},{q}_{{{{{{{{\mathcal{S}}}}}}}}(i,{{{{{{{\mathcal{C}}}}}}}}(i))},{q}_{{{{{{{{\mathcal{C}}}}}}}}(i)},{y}^{l},w)\right.},$$

where ${{{{{{{\mathcal{P}}}}}}}}(i)$ refers to the set of parent nodes, ${{{{{{{\mathcal{C}}}}}}}}(i)$ refers to the set of child nodes, and ${{{{{{{\mathcal{S}}}}}}}}(i)$ refers to the set of spouse nodes that share the same child nodes with *i*. *T* is a nonlinear function that is determined by the prior of *i*, weights of edges associated with node *i*, and posteriors of the parent, child, and spouse nodes of *i* as follows:

6 $$T(\cdot )\,=	\mathop{\sum}\limits_{\begin{array}{c}k\in {{{{{\mathcal{P}}}}}}(i)\\ \nu \in \{0,1\}\end{array}}\left\{{(-1)}^{\nu }{q}_{k}f\left({(-1)}^{\nu }({w}_{0i}+{w}_{ki})+\frac{{w}_{{\epsilon }_{i}}^{2}}{2}\right)+{(-1)}^{\nu }(1-{q}_{k})f\left({(-1)}^{\nu }{w}_{0i}+\frac{{w}_{{\epsilon }_{i}}^{2}}{2}\right)\right\}\\ 	+\mathop{\sum}\limits_{\begin{array}{c}k\in {{{{{\mathcal{C}}}}}}(i)\\ k\ne y\\ \nu \in \{0,1\}\end{array}}\left\{{(-1)}^{\nu }{q}_{k}f\left({(-1)}^{\nu }({w}_{0i}+{w}_{ik})+\frac{{w}_{{\epsilon }_{k}}^{2}}{2}\right)+{(-1)}^{\nu }(1-{q}_{k})f\left({(-1)}^{\nu }{w}_{0i}+\frac{{w}_{{\epsilon }_{k}}^{2}}{2}\right)\right\}\\ 	-\frac{2{\sum }_{m\in {{{{{\mathcal{S}}}}}}(i,y)}{w}_{iy}{w}_{my}{q}_{m}}{2{w}_{{\epsilon }_{y}}^{2}}+\frac{(2\log y-2{w}_{0y}-{w}_{iy}^{2}){w}_{iy}}{2{w}_{{\epsilon }_{y}}^{2}},$$

where $f(x)=\log (1+\exp (x))$. The logistic function ensures that the posterior is between 0 and 1. The update for unobserved node *i* depends only on the states of its parents, children, and spouses.

In the maximization step, we conduct stochastic gradient descent updates to estimate the optimal weights using a mini-batch of data randomly sampled from different locations. Stochastic variational inference is designed here to accelerate the computational process over a large-scale high-resolution map. The edge weights at the iteration *t* + 1 are therefore updated as:

7 $${{{{{{{{\bf{w}}}}}}}}}^{(t+1)}={{{{{{{{\bf{w}}}}}}}}}^{(t)}+\rho {{{{{{{\mathcal{A}}}}}}}}\nabla {{{{{{{{\bf{L}}}}}}}}}^{(t)}({{{{{{{\bf{w}}}}}}}})$$

where *ρ* controls the learning rate, and ${{{{{{{\mathcal{A}}}}}}}}$ is a preconditioner. Here, ${{{{{{{\mathcal{A}}}}}}}}$ is set up as the identity matrix to accelerate convergence to high-likelihood models. In each iteration, we first randomly sample a mini-batch of locations from the given map. Then, the expectation step and maximization step are implemented to update the posterior estimations and the global weight parameters. As the model converges, the optimal posteriors of landslide, liquefaction, and building damage at each location are estimated.

#### Local pruning for accelerating the computation

Computational efficiency is another key element determining the applicability of multi-hazard estimation in real-world scenarios. We observed that real-world causal graph tends to be more sparse in many locations: only a subset of nodes are active for each location. For example, if there is no building footprint, the building damage node will be inactive. We also observed that when the slope is steep, liquefaction has a low probability to co-occur with landslide^61^. Therefore, when the prior landslide probability is significantly higher than the prior liquefaction estimation (e.g., the difference between prior landslide and liquefaction is higher than the median of the overall difference distribution between prior landslide and liquefaction estimations), we can treat the liquefaction node as inactive. For inactive nodes, the posterior probability of their ancestor nodes is typically very small. Moreover, these inactive nodes have little influence on parameter updates on the graph as the gradients of edge weights during updating process are proportional to the activation probabilities. According to the above intuition, we developed a local pruning strategy to remove these irrelevant subsets of the graph, while still retaining the nodes that contain information crucial to the subsequent parameter update. Specifically, we construct a location-specific local model as follows:

1. Select ${{{{{{{{\mathcal{O}}}}}}}}}_{l}^{+}$, the set of active nodes for the location *l*.
2. Select ${{{{{{{{\mathcal{H}}}}}}}}}_{l}$, the parents of nodes in ${{{{{{{{\mathcal{O}}}}}}}}}_{l}^{+}$. We do explicit variational inference updates only for this subset of nodes.
3. Link the selected node with causal dependencies.

Given each location, we conduct local pruning first to obtain a local model and then adapt stochastic variational inference over the local model. Our derived theoretically sound variational bound holds on any similar network structure and variable assumptions as our network. Therefore, the stochastic variational inference algorithm can be easily adapted by setting the posterior *q*_*i*_ of inactive nodes *i* as zero. The combination of stochastic variational inference and local pruning dramatically reduces the computational cost and memory demands.

#### Prior geospatial models

The prior landslide and liquefaction models we used for comparison in the results are geospatial models from existing USGS ground failure products. As part of the Earthquake Hazards Program, the USGS developed a ground-failure earthquake product to augment the Prompt Assessment of Global Earthquakes for Response system^5^. Following major earthquakes worldwide, this product provides near real-time maps of earthquake-induced landslide and liquefaction probabilities. Both the liquefaction and landslide maps are derived from models that utilize ground-shaking intensity as an input, which allows the maps to be rapidly generated, but do not take into account information from remotely sensed images. Ground-motion inputs are taken from ShakeMaps, which, in turn, are derived from instrumental recordings, ground-motion models, and site conditions estimated from the shallow shear velocity. Additional inputs related to liquefaction vulnerability include mean annual precipitation, distance from the coast, distance from rivers, and water table depth.

#### Evaluation metrics

We mainly utilize the ROC (receiver operating characteristic) curve and Cross-entropy loss to evaluate and compare the performance of our system.

#### ROC curves

are a visualization method to represent the ability of a binary classifier system as its discrimination threshold is varied^62^. Both our system and prior models mainly output the probability estimation of landslide, liquefaction, or building damage. In real-world applications, a threshold is often applied to the probability to divide the estimates into “there exists damage” and “no damage”. As the threshold varies from event to event, it would be unfair to directly set up a fixed threshold to evaluate the system performance. In contrast, the ROC curve visualizes how the system performance changes under a varying threshold ranging between 0 and 1, which is fairer. The ROC curve is created by plotting the true positive rate (TPR) against the false positive rate (FPR) at various threshold settings. Given a binary classification problem, label 1 represents that a damage has occurred in the location. We define the true positive (TP) as that both predicted label and true label are 1, i.e., both prediction model and ground truth observations that the damage has occurred. Similarly, true negative (TN) refers to that both prediction model and ground truth observations indicate that no damage has occurred. In contrast, if the predicted label is 1 but true label is 0, i.e., the prediction model estimates the damage has occurred, but the damage is not observed in the ground truth data, we define it as a false positive (FP) prediction and otherwise false negative (FN). The true positive rate (TPR) is also known as sensitivity, recall, or probability of detection, and can be calculated as $\frac{\#{{{{{{{\rm{TP}}}}}}}}}{\#{{{{{{{\rm{TP}}}}}}}}+\#{{{{{{{\rm{FN}}}}}}}}}$. The false positive rate (FPR) is also known as the probability of false alarm and can be calculated as $\frac{\#{{{{{{{\rm{FP}}}}}}}}}{\#{{{{{{{\rm{FP}}}}}}}}+\#{{{{{{{\rm{TN}}}}}}}}}$.

#### Cross-entropy loss

is one of the most widely used loss functions^63^ to measure the performance of a classification model whose output is a probability value between 0 and 1. It is designed as the mutual information between two probability distributions, mostly one discrete distribution and one continuous distribution, over the same underlying set of events. Mathematically, for binary classification models, a cross-entropy loss (CEL) is defined as below:

8 $${{{{{{{\rm{CEL}}}}}}}}=-\frac{1}{N}\mathop{\sum }\limits_{n=1}^{N}[{z}_{n}\log {P}_{n}+(1-{z}_{n})\log (1-{P}_{n})],$$

where *z*_*n*_ ∈ {0, 1} represents the true label, *z*_*n*_ = 1 represents that damage occurred, and *P*_*n*_ represents the predicted probability of the damage. As the predicted probability diverges from the true label, the cross-entropy loss increases. For example, predicting one pixel’s landslide probability of 0.001 while actual landslides are observed in the corresponding location will yield a high loss value, meaning that the prediction model has high uncertainties about the landslides occurrence variable. Ideally, a perfect model would have a cross-entropy loss of 0. Therefore, smaller cross-entropy loss often means a more accurate model that can better predict the true label distribution.

## Supplementary information

Supplementary information

## Data Availability

For the four earthquake events presented, the building damage, posterior landslide, and posterior liquefaction models generated in this study have been deposited in the Zenodo database under accession code 10.5281/zenodo.7319726^64^. For the input datasets, the original sources of prior models and building footprints are publicly available at https://earthquake.usgs.gov/^28–31^ and http://download.geofabrik.de/^23^, respectively. The DPM for the 2020 Puerto Rico earthquake is also publicly available at https://aria-share.jpl.nasa.gov/20200106-Puerto_Rico_EQ/DPM/^42^. The updated DPMs for other earthquake events are under restricted access because these are not made publicly available at ARIA website, access can be obtained by contacting S.X. Post-processed DPMs, prior models, and building footprints to be used as inputs to our code have also been deposited in the Zenodo database under accession code 10.5281/zenodo.7319726^64^. Some of the original sources of ground truth datasets are publicly available through the following links: (1) 2016 Central Italy earthquake: landslide (https://www.sciencebase.gov/catalog/item/5b6b2201e4b006a11f7795c3)^41,48^; (2) 2018 Hokkaido, Japan earthquake: landslide (https://www.gsi.go.jp/common/000204728.zip)^47^; (3) 2019 Ridgecrest, California earthquake: landslide, liquefaction, and building damage (https://www.designsafe-ci.org/data/browser/public/designsafe.storage.published/PRJ-2440)^17,57,59^; and (4) 2020 Puerto Rico earthquake: landslide, liquefaction, and building damage (https://www.sciencebase.gov/catalog/item/5eb5b9dc82ce25b5135ae83a)^40^. The building damage ground truth dataset^54^ after the 2016 Central Italy earthquake and the some landslide and liquefaction ground truth data^58^ are under restricted access because these are not made publicly available by the original authors, access can be obtained by contacting S.X.

## Code availability

The source codes for the implementation of the joint natural hazards estimation workflow including the scripts for evaluation metrics are publicly available online under accession code 10.5281/zenodo.7319726^64^.
