# Supplementary material for: Seismic multi-hazard and impact estimation via causal inference from satellite imagery
Source: Nat Commun. 2022 Dec 17;13:7793. doi: 10.1038/s41467-022-35418-8 (PMC9758197; doi:10.1038/s41467-022-35418-8)
Supplement: Supplementary file 1 — Supplementary information [file 41467_2022_35418_MOESM1_ESM.pdf]

Inventory of Supporting Information

Manuscript #: NCOMMS-22-00675B
Corresponding author name: Susu Xu

Supplementary figure:

Figure #	Figure caption
Supplementary Fig. 1	Geospatial prior and posterior estimation models of the 2016 Central Italy earthquake<br>**a** Damage proxy map (30-meter resolution) with rectangular extents of each focused area. **b** Prior landslide model. c Posterior landslide model. **d** Prior liquefaction model. **e** Posterior liquefaction model. **f** Building footprint map. **g** Posterior building damage model.
Supplementary Fig. 2	Geospatial prior and posterior estimation models of the 2019 Ridgecrest earth-<br>quake. **a** Damage proxy map (30-meter resolution) with rectangular extents of each focused area. **b** Prior landslide model. c Posterior landslide model. **d** Prior Liquefaction model. **e** Posterior liquefaction model. **f** Building footprint map. **g** Posterior building damage model.
Supplementary Fig. 3	Geospatial prior and posterior estimation models of the 2018 Hokkaido, Japan<br>earthquake. **a** Damage proxy map (5-meter resolution) with rectangular extents of each focused area. **b** Prior landslide model. **c** Posterior landslide model. **d** Prior landslide model with ground truth observation. **e** Posterior landslide model with ground truth observation. **f** Prior liquefaction model. **g** Posterior liquefaction model. h Building footprint map. **i** Posterior building damage model.
Supplementary Fig. 4	**a** Visualization of quantified causal graph for the 2016 Central Italy earthquake. **b** Visualization of quantified causal graph for the 2019 Ridgecrest, CA earthquake. **c** Estimated building damage probability distribution within the landslide region (transparent white) and the liquefaction zone (colored blue) for the 2016 Central Italy earthquake. **d** Estimated building damage probability distribution within the landslide area (transparent white) and the liquefaction zone (colored blue) for the 2019 Ridgecrest, CA earthquake
Supplementary Fig. 5	**a** Posterior building damage probability estimations at different ground truth dam-<br>age states for the 2020 Puerto Rico earthquake. **b** Posterior building damage probability estimation at<br>different ground truth damage states for the 2016 Central Italy earthquake.

Supplementary table:

Figure #	Figure caption
Supplementary Table 1	Notations for Random Variables in the Causal Bayesian Network Formulation
Supplementary Table 2	Mean and standard deviation of causal coefficients learned from the causal Bayesian network on multiple events and DPMs with different resolutions. (BD: Building Damage, LS: Landslide, L: Liquefaction, GS: Ground Shaking.)
Supplementary Table 3	Summary of ground truth information for each earthquake event.

Supplementary Discussion: Available below.

Supplementary Information

1 Supplementary Discussion

The Supplementary Fig. 1 visualizes our model performance in shaken regions of the 2016 Central Italy earthquake for joint landslide (Supplementary Fig. 1c), liquefaction (Supplementary Fig. 1e), and building damage estimation (Supplementary Fig. 1g), compared with the prior landslide model (Supplementary Fig. 1b), prior liquefaction model (Supplementary Fig. 1d), and building footprint map (Supplementary Fig. 1f). Compared to prior landslide models, our system achieves 13.83% improvement in AUC of the landslide ROC curve, enhancing the true positive rate from 80% to 94.17% with 62.50% false positive rate. Comparing Fig. 1d and e, our model significantly reduces the false positive rate of liquefaction estimation while retaining high probability in the water-body areas. Moreover, as shown in Supplementary Fig. 1g, without any prior information or model about the building damage, our building damage posterior matches with the spatial distribution of building damage, indicating that the causal Bayesian network provides a reasonable quantification of the underlying causal dependencies. Supplementary Fig. 4a visualizes the learned causal effects over the causal graph. The width of edges represents the causal effect importance. The results show that most building damage captured by satellite images are mainly caused by landslides. In Fig. 4c, building damage estimates in the landslide zone tend to have higher probabilities than the estimates in the liquefaction zone.

The Supplementary Fig. 2 presents our model performance in the 2019 Ridgecrest, CA earthquake for joint landslide (Supplementary Fig. 2c), liquefaction (Supplementary Fig. 2e), and building damage estimates (Supplementary Fig. 2g), as well as the prior landslide model (Supplementary Fig. 2b), prior liquefaction model (Supplementary Fig. 2d), and building footprint (Supplementary Fig. 2f). Compared to prior models, our system improves the AUC of landslide and liquefaction ROC curves by 8.68% and

10.61%, and reduces the cross-entropy loss by 24.06% and 39.16% compared to the prior models, respectively. Comparing Supplementary Fig. 2d and e, our model significantly reduces the false positive rate of liquefaction estimates while increasing probabilities in the areas with more observed liquefaction.

The Supplementary Fig. 3 shows the estimation results from DPMs with 5-m resolution captured in the 2018 Hokkaido, Japan earthquake for joint landslide (Supplementary Fig. 2c and e), liquefaction (Supplementary Fig. 2g), and building damage estimates (Supplementary Fig. 2i), as well as the prior landslide model (Supplementary Fig. 2b and d), prior liquefaction model (Supplementary Fig. 2f), and building footprint (Supplementary Fig. 2h). Compared to 30-m DPMs, 5-m DPMs (Supplementary Fig. 2a) contain more noise from anthropogenic environment changes, such as changes to farmlands. Overall, our posterior landslide estimates reduce the false positive rate significantly while retaining accurate predictions of true landslide occurrences. It can be noted that compared to 30-m DPMs, the resolutions of 5-m DPM based estimations are further refined to provide more specific locations of landslides, liquefaction, and building damage. Notably, this is the first regional building damage distribution map with 5-m resolution, which has comparable size to a typical building dimension and shows the potential to be directly used for further large-scale risk assessment.

The Supplementary Fig. 4b visualizes the causal graph obtained in our analysis. In the Ridgecrest earthquake, building damage occurred in the areas dominated by the impacts of liquefaction and ground shaking. The estimates of building damage posteriors, shown in Fig. 4d, also indicate that building damage probabilities in the liquefaction zone are significantly higher than in the landslide zone. The results match with the observations that during the 2019 Ridgecrest earthquake, liquefaction in the town of Trona induced one large lateral spread and caused severe damage to around 30 square blocks[1]. Supplementary Table 2 further provides the detailed values of causal coefficients and their standard deviations obtained from multiple experiments.

Supplementary Fig. 5 presents the cumulative distribution function for our posterior building damage estimations at each damage state in the 2020 Puerto Rico and 2016 Central Italy earthquakes. Notably, the posterior building damage are jointly estimated with landslide and liquefaction posteriors by incorporating the causal dependencies, and no prior building damage model nor any ground truth data are used. It can be found that our estimated building damage distributions present different patterns at each damage state. In Supplementary Fig. 5a and b, as the damage state becomes more severe, the posterior building damage estimation tends to assign higher probability. At this stage, our posterior building damage map result can be viewed from the regional perspective where we were able to highlight areas that need attention. Alternative existing seismic building damage detection and quantification methods tend to suffer from low accuracy and resolution, low scalability, or long latency times[2,3]. Our approach has been demonstrated to provide a promising way for generating a regional high-resolution building damage map rapidly without the need for any prior model. All-in-all, our strategy points to a new research and application realm for the estimation of near-real-time seismic hazards and their impacts.

We also compared the performance of our building damage detection with building fragility function dedicatedly developed for Puerto Rico[4] and for Italy[5] as well as the fragility function for Ridgecrest developed by HAZUS. Note that since fragility function is not geospatial approach, we did not present the ROC curves in the Figure 2. Our experiments showed that our system achieved upto 35.43% AUC improvement compared to the fragility function-based method. Note that not only is the building inventory information is much more complete in the United States compared to other countries, the building fragility functions in HAZUS are also much more accurate for the U.S. Therefore we have relatively high AUC for fragility function in the Ridgecrest earthquake. But this is not often the case in other nations. Moreover, since building type and pre-disaster conditions are not known, the fragility function may be very inaccurate for areas with a wide range of building structures and conditions. Our model targets on the earthquake events all

over the world and achieves constantly better performance than fragility function by combining the DPM information. In the future, our system is flexible to evolve to accommodate improved building inventories and fragilities worldwide as they are developed.

2 Supplementary Figures

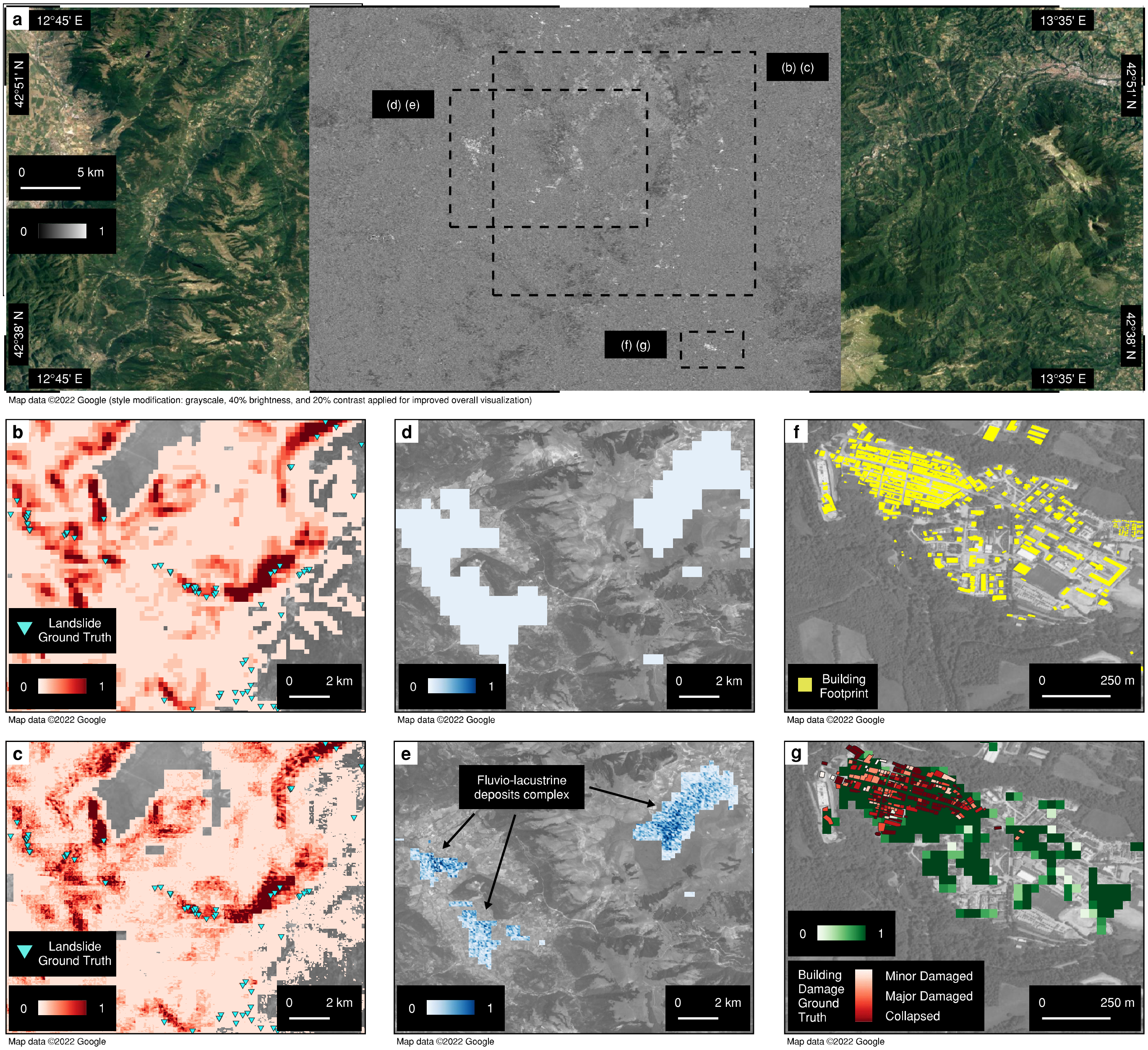

Supplementary Fig. 1. Geospatial prior and posterior estimation models of the 2016 Central Italy earthquake. **a** Damage proxy map (30-meter resolution) with rectangular extents of each focused area. **b** Prior landslide model. **c** Posterior landslide model. **d** Prior liquefaction model. **e** Posterior liquefaction model. **f** Building footprint map. **g** Posterior building damage model.

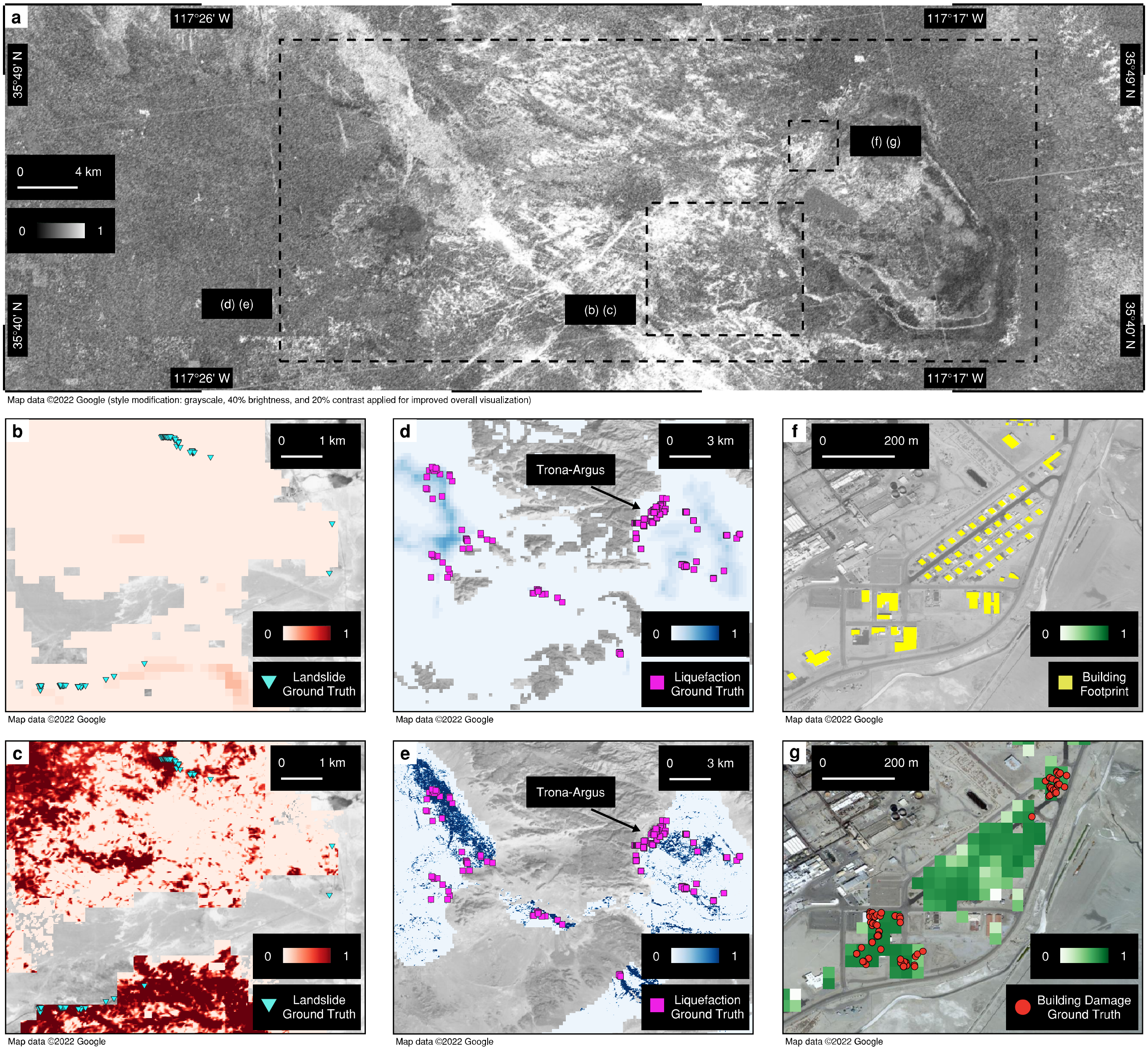

Supplementary Fig. 2. Geospatial prior and posterior estimation models of the 2019 Ridgecrest earthquake. **a** Damage proxy map (30-meter resolution) with rectangular extents of each focused area. **b** Prior landslide model. **c** Posterior landslide model. **d** Prior liquefaction model. **e** Posterior liquefaction model. **f** Building footprint map. **g** Posterior building damage model.

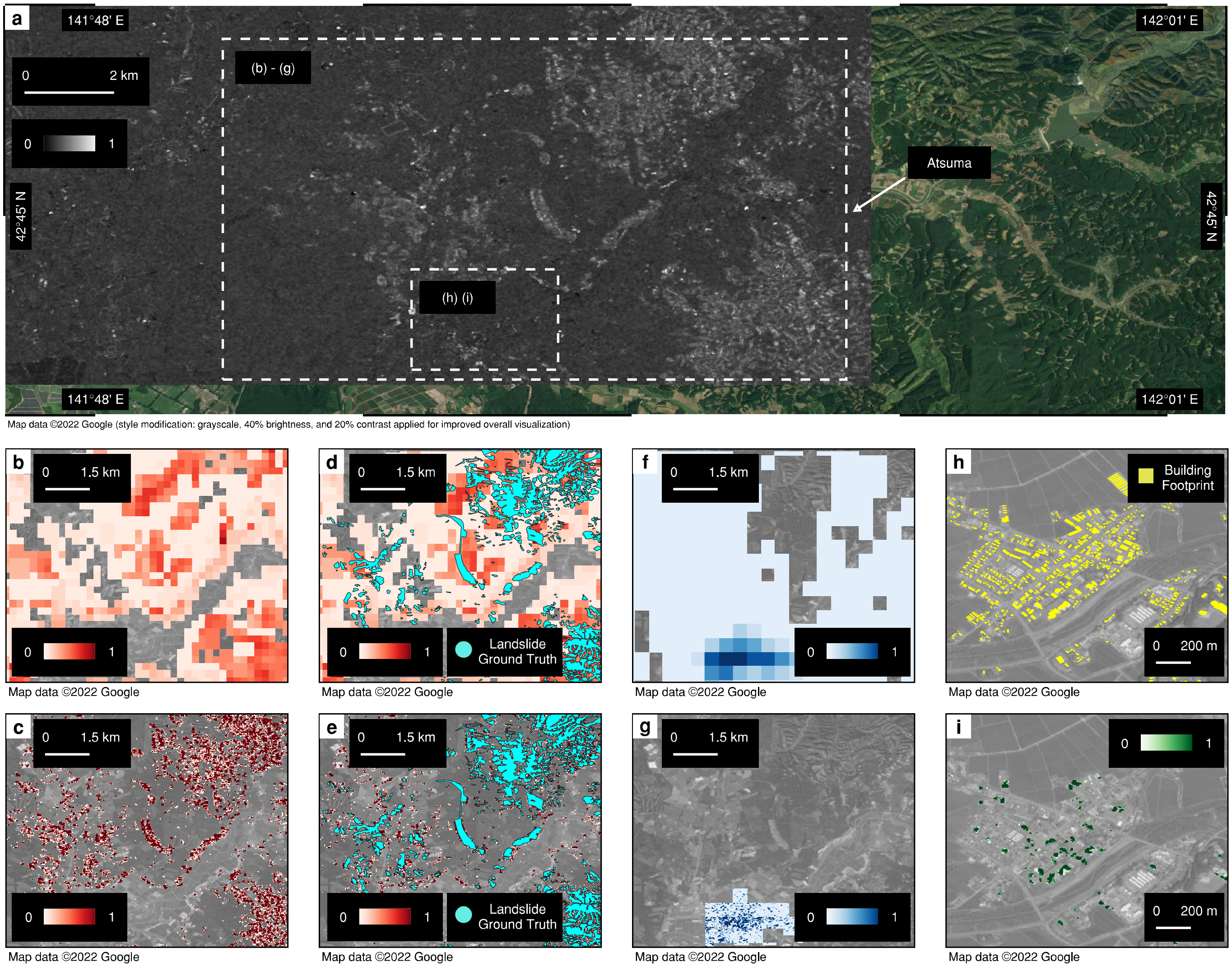

Supplementary Fig. 3. Geospatial prior and posterior estimation models of the 2018 Hokkaido, Japan earthquake. **a** Damage proxy map (5-meter resolution) with rectangular extents of each focused area. **b** Prior landslide model. **c** Posterior landslide model. **d** Prior landslide model with ground truth observation. **e** Posterior landslide model with ground truth observation. **f** Prior liquefaction model. **g** Posterior liquefaction model. **h** Building footprint map. **i** Posterior building damage model.

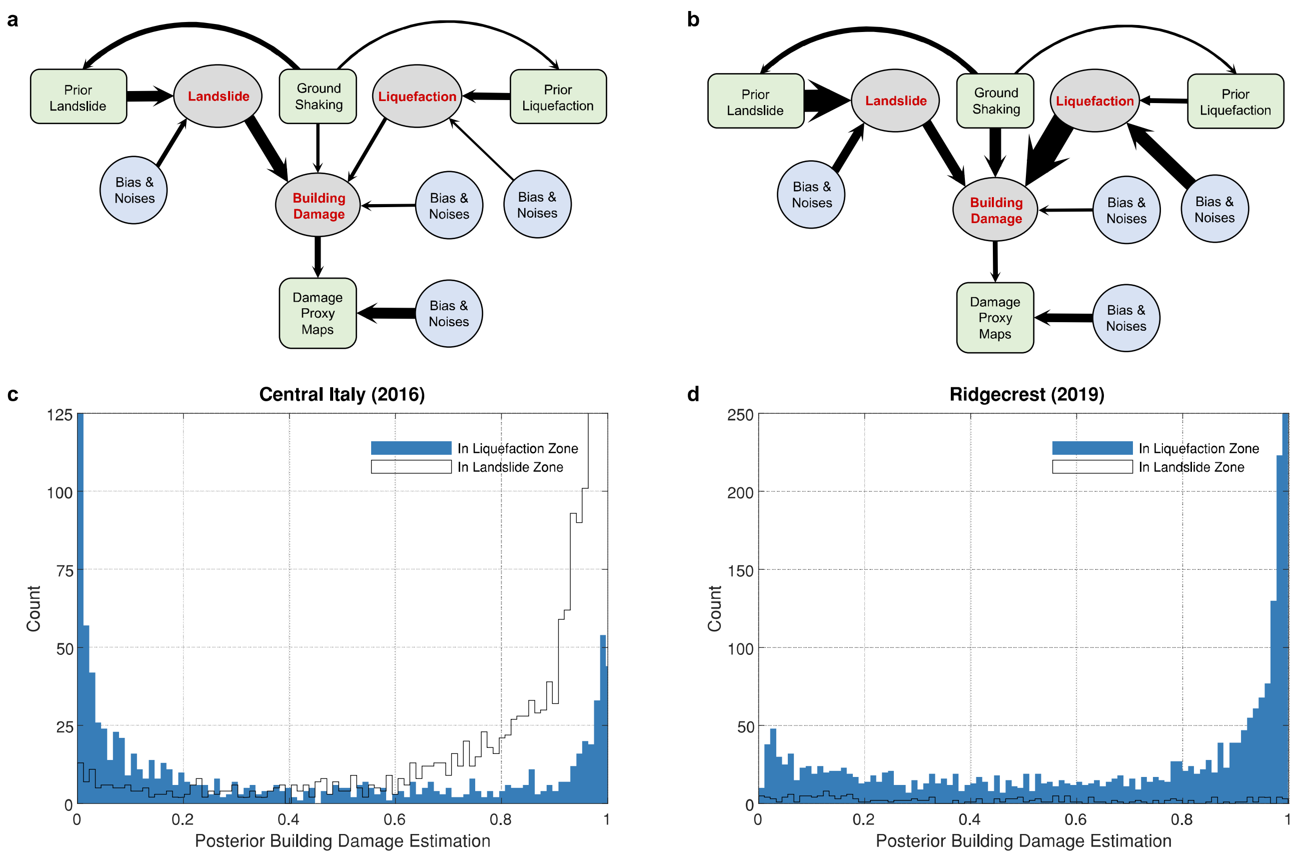

Supplementary Fig. 4. **a** Visualization of quantified causal graph for the 2016 Central Italy earthquake. **b** Visualization of quantified causal graph for the 2019 Ridgecrest, CA earthquake. **c** Estimated building damage probability distribution within the landslide region (transparent white) and the liquefaction zone (colored blue) for the 2016 Central Italy earthquake. **d** Estimated building damage probability distribution within the landslide area (transparent white) and the liquefaction zone (colored blue) for the 2019 Ridgecrest, CA earthquake.

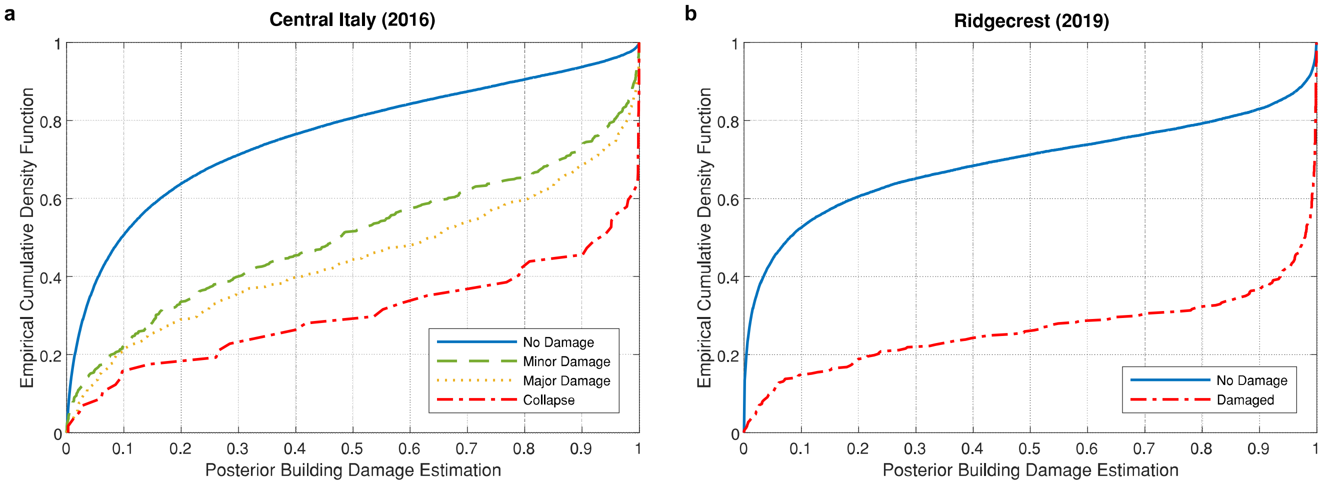

Supplementary Fig. 5. a Posterior building damage probability estimations at different ground truth damage states for the 2020 Puerto Rico earthquake. **b** Posterior building damage probability estimation at different ground truth damage states for the 2016 Central Italy earthquake.

3 Supplementary Tables

Supplementary Table 1. Notations for Random Variables in the Causal Bayesian Network Formulation

Symbol	Description	Probability Distribution	Observed or not?
LS	Landslide	Bernoulli	No
LF	Liquefaction	Bernoulli	No
BD	Building damage	Bernoulli	No
x_0	Intercept	Bernoulli	No
ϵ	noise	Standard Normal	No
α_{LS}	Prior LS estimation	Logit-Normal	Yes
α_{LF}	Prior LF estimation	Logit-Normal	Yes
DPM	DPM value	Log-Normal	Yes

Supplementary Table 2. Mean and standard deviation of causal coefficients learned from the causal Bayesian network on multiple events and DPMs with different resolutions. (BD: Building Damage, LS: Landslide, L: Liquefaction, GS: Ground Shaking.)

	Puerto Rico	Japan - 30m	Japan - 5m	Ridgecrest	Central Italy
BD→DPMs	$\mathbf{0.210}(\pm\mathbf{0.0116})$	$0.085(\pm0.0162)$	$0.230(\pm0.2772)$	$0.398(\pm0.0037)$	$\mathbf{0.532}(\pm\mathbf{0.0035})$
LS→DPMs	$0.077(\pm0.0179)$	$\mathbf{0.363}(\pm\mathbf{0.0022})$	$\mathbf{0.344}(\pm\mathbf{0.0006})$	$0.372(\pm0.0002)$	$0.048(\pm0.0283)$
LF→DPMs	$0.187(\pm0.0375)$	$0.053(\pm0.0272)$	$-0.083(\pm0.0055)$	$\mathbf{0.446}(\pm\mathbf{0.0003})$	$0.061(\pm0.0032)$
Bias→DPMs	$-0.728(\pm0.0016)$	$-0.798(\pm0.0016)$	$-1.092(\pm0.0023)$	$-0.720(\pm0.0001)$	$-1.057(\pm0.0020)$
GS→BD	$0.032(\pm0.1817)$	$0.732(\pm0.3692)$	$0.722(\pm0.1088)$	$1.200(\pm0.0001)$	$0.270(\pm0.0607)$
LS→BD	$0.527(\pm0.3054)$	$\mathbf{0.848}(\pm\mathbf{0.1282})$	$\mathbf{1.272}(\pm\mathbf{1.401})$	$1.599(\pm0.3741)$	$\mathbf{0.966}(\pm\mathbf{0.5751})$
LF→BD	$\mathbf{1.718}(\pm\mathbf{0.1163})$	$0.432(\pm0.1608)$	$0.217(\pm0.779)$	$\mathbf{2.060}(\pm\mathbf{0.1510})$	$0.345(\pm0.1568)$
GS→LS	$1.406(\pm0.2830)$	$0.703(\pm0.0125)$	$0.392(\pm0.0056)$	$5.628(\pm0.0014)$	$1.485(\pm0.6598)$
Bias→LS	$-0.073(\pm0.0828)$	$-0.153(\pm0.0324)$	$-2.176(\pm0.0028)$	$1.098(\pm0.0011)$	$-0.386(\pm0.4250)$
GS→LF	$0.259(\pm0.1493)$	$0.415(\pm0.0614)$	$0.384(\pm0.0502)$	$0.063(\pm0.0002)$	$0.013(\pm0.0934)$
Bias→LF	$-0.577(\pm0.3207)$	$-0.491(\pm0.0908)$	$-0.397(\pm0.0765)$	$-2.074(\pm0.0004)$	$0.617(\pm0.1043)$

Supplementary Table 3. Ground truth dataset summary.

Event	Datasets	Availability	Further Information
3*2016 Central Italy earthquake	Landslide	Yes	Points of landslide locations[6,7]
	Liquefaction	No	There is no available geotagged information for the limited extent of the damage proxy map.
	Building Damage	Yes	Points of building damage[8] (with three levels of severity) locations
3*2018 Hokkaido, Japan earthquake	Landslide	Yes	Polygons identifying the extent of landsliding[9]
	Liquefaction	No	The liquefaction investigation does not cover the extent of the damage proxy map.
	Building Damage	No	There is no available geotagged building damage information for the extent of the damage proxy map.
3*2019 Ridgecrest, California earthquake	Landslide	Yes	Points of landslide locations[10–13]
	Liquefaction	Yes	Points of liquefaction locations[10–13]
	Building Damage	Yes	Points of building damage locations[10–13]
3*2020 Puerto Rico earthquake	Landslide	Yes	Points of landslide locations and polygons identifying the extent of landsliding[14]
	Liquefaction	Yes	Points of liquefaction locations[14]
	Building Damage	Yes	Points of building damage (with three levels of severity) locations[14]

References

1. Jibson, R. W. Types and areal distribution of ground failure associated with the 2019 Ridgecrest, California, earthquake sequence. *Bulletin of the Seismological Society of America* **110**, 1567–1578 (2020).

2. Matsuoka, M. & Nojima, N. Building damage estimation by integration of seismic intensity information and satellite L-band SAR imagery. *Remote Sensing* **2**, 2111–2126 (2010).

3. Hasik, V., Chhabra, J. P., Warn, G. P. & Bilec, M. M. Review of approaches for integrating loss estimation and life cycle assessment to assess impacts of seismic building damage and repair. *Engineering Structures* **175**, 123–137 (2018).

4. Mieses, L. A., López, R. R. & Saffar, A. Development of fragility curves for medium rise reinforced concrete shear wall residential buildings in puerto rico. *Mecánica Computacional* 2712–2727 (2007).

5. Del Gaudio, C. *et al.* Empirical fragility curves from damage data on rc buildings after the 2009 l'aquila earthquake. *Bulletin of Earthquake Engineering* **15**, 1425–1450 (2017).

6. Martino, S., Prestininzi, A. & Romeo, R. Earthquake-induced ground failures in Italy from a reviewed database. *Natural Hazards and Earth System Sciences* **14**, 799–814 (2014).

7. Schmitt, R. G. *et al.* An open repository of earthquake-triggered ground-failure inventories. Tech. Rep., US Geological Survey (2017).

8. Fiorentino, G. *et al.* Damage patterns in the town of Amatrice after August 24th 2016 Central Italy earthquakes. *Bulletin of earthquake engineering* **16**, 1399–1423 (2018).

9. Geospatial Institute of Japan. Slope collapse and sedimentation distribution map associated with the 2018 Hokkaido Eastern Iburi Earthquake (around Atsuma Town, Hokkaido), Technical Data D1-No.991. Available at

www.gsi.go.jp/BOUSAI/H30-hokkaidoiburi-east-earthquake-index.html (2021/01/24).

10. Zimmaro, P. *et al.* Liquefaction and related ground failure from july 2019 ridgecrest earthquake sequence. *Bulletin of the Seismological Society of America* **110**, 1549–1566 (2020).

11. Brandenberg, S. J. *et al.* Preliminary report on engineering and geological effects of the July 2019 Ridgecrest earthquake sequence. Tech. Rep., Geotechnical Extreme Event Reconnaissance Association (2019).

12. Jibson, R. W. Types and areal distribution of ground failure associated with the 2019 Ridgecrest, California, earthquake sequence. *Bulletin of the Seismological Society of America* **110**, 1567–1578 (2020).

13. Brandenberg, S. *et al.* GEER field reconnaissance, Ridgecrest, CA earthquake sequence, July 4 and 5, 2019. Available at https://doi.org/10.17603/DS2-VPMV-5B34 (2021/01/24).

14. Allstadt, K. *et al.* Field observations of ground failure triggered by the 2020 Puerto Rico earthquake sequence. U.S. Geological Survey data release. Available at https://doi.org/10.5066/P96QNFMB (2021/01/24).
